# BH3 Mimetics for the Treatment of B-Cell Malignancies—Insights and Lessons from the Clinic

**DOI:** 10.3390/cancers12113353

**Published:** 2020-11-12

**Authors:** Victor S. Lin, Zhuo-Fan Xu, David C. S. Huang, Rachel Thijssen

**Affiliations:** 1 The Walter and Eliza Hall Institute of Medical Research, 1G Royal Parade, 3052 Parkville, Australia; lin.v@wehi.edu.au (V.S.L.); xu.zh@wehi.edu.au (Z.-F.X.); huang_d@wehi.edu.au (D.C.S.H.); 2 Faculty of Medicine, Dentistry and Health Sciences, University of Melbourne, 3000 Melbourne, Australia; 3 School of Medicine, Tsinghua University, 30 Shuangqing Road, Haidian District, Beijing 100084, China; 4 Department of Medical Biology, University of Melbourne, 3000 Melbourne, Australia

**Keywords:** BH3 mimetics, venetoclax, apoptosis, BCL2, MCL1, BCLxL, B-cell malignancies, leukemia, lymphoma, myeloma

## Abstract

**Simple Summary:**

B-cell malignancies, including chronic lymphocytic leukemia (CLL), non-Hodgkin lymphoma (NHL), and plasma cell dyscrasias, are significant contributors to cancer morbidity and mortality worldwide. The pathogenesis of many B-cell malignancies involves perturbations in the intrinsic pathway of apoptosis that allow cells to evade cell death. BH3 mimetics represent a class of anti-cancer agents that can restore the ability of cancer cells to undergo apoptosis. Venetoclax, a recently approved BH3 mimetic, has transformed the therapeutic landscape for CLL. Other BH3 mimetics are currently under development. This review summarizes the available data on existing BH3 mimetics and highlights both the rapidly expanding role of BH3 mimetics in the treatment of B-cell malignancies and the clinical challenges of their use.

**Abstract:**

The discovery of the link between defective apoptotic regulation and cancer cell survival engendered the idea of targeting aberrant components of the apoptotic machinery for cancer therapy. The intrinsic pathway of apoptosis is tightly controlled by interactions amongst members of three distinct subgroups of the B-cell lymphoma 2 (BCL2) family of proteins. The pro-survival BCL2 proteins prevent apoptosis by keeping the pro-apoptotic effector proteins BCL2-associated X protein (BAX) and BCL2 homologous antagonist/killer (BAK) in check, while the BH3-only proteins initiate apoptosis by either neutralizing the pro-survival BCL2 proteins or directly activating the pro-apoptotic effector proteins. This tripartite regulatory mechanism is commonly perturbed in B-cell malignancies facilitating cell death evasion. Over the past two decades, structure-based drug discovery has resulted in the development of a series of small molecules that mimic the function of BH3-only proteins called the BH3 mimetics. The most clinically advanced of these is venetoclax, which is a highly selective inhibitor of BCL2 that has transformed the treatment landscape for chronic lymphocytic leukemia (CLL). Other BH3 mimetics, which selectively target myeloid cell leukemia 1 (MCL1) and B-cell lymphoma extra large (BCLxL), are currently under investigation for use in diverse malignancies. Here, we review the current role of BH3 mimetics in the treatment of CLL and other B-cell malignancies and address open questions in this rapidly evolving field.

## 1. Introduction

Cell death is an important biological event that enables the clearance of unwanted or injured cells. Broadly speaking, cells can die in a highly regulated (or “programmed”) fashion through the process of apoptosis or in an uncontrolled manner through the process of necrosis [1]. First described by Kerr, Wyllie, and Currie in 1972, apoptosis, which can be triggered by a range of internal and external stimuli, is characterized morphologically by shrinkage of the nucleus and mitochondria, encasement of cellular contents in membrane-bound apoptotic bodies, and rapid clearance of apoptotic bodies by adjacent phagocytes [2]. In contrast, necrosis, which usually results from acute cell trauma, is marked by swelling of the mitochondria, rupture of the plasma membrane, and spillage of inflammatory cellular contents into the surrounding tissue. Apoptosis has been found to play an essential role in the achievement of normal development [3,4] and the maintenance of tissue homeostasis [5,6]. Defects in the regulation of apoptosis can result in a number of diseases, including cancer [7].

**Figure 1:**
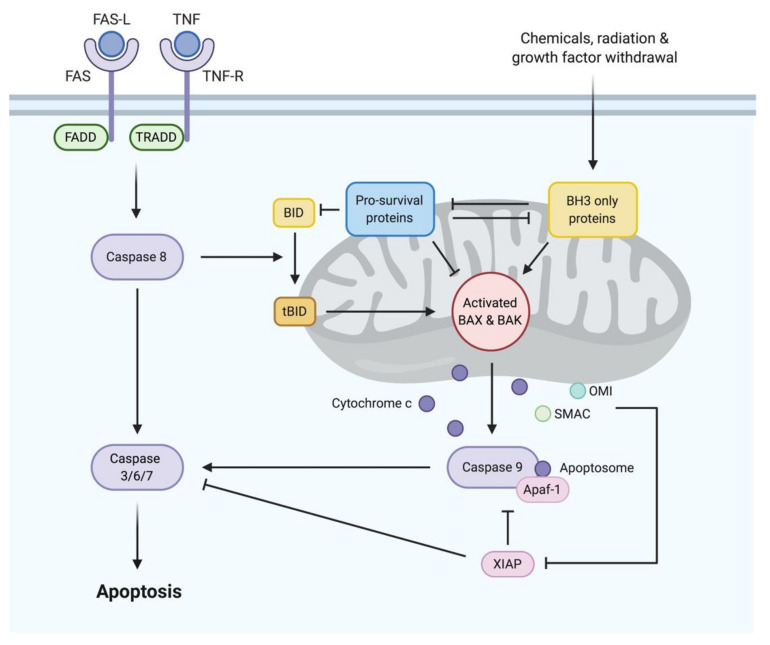
The extrinsic and intrinsic pathways to apoptosis. The extrinsic pathway is initiated when death receptor ligands (e.g., first apoptosis signal ligand [FAS-L], tumor necrosis factor (TNF)) bind to their cognate death receptors (e.g., first apoptosis signal [FAS], TNF receptor [TNFR]) on the plasma membrane, resulting in activation of caspase 8 via FAS-associated death domain protein (FADD) with or without TNFR-associated death domain protein (TRADD). The intrinsic pathway is triggered when diverse stress signals (e.g., DNA damage, growth factor deprivation) activate pro-apoptotic BH3-only proteins, which carry out their pro-apoptotic function by neutralizing pro-survival B-cell lymphoma 2 (BCL2) family proteins or, when these pro-survival proteins are saturated or absent, by directly activating BCL-associated X protein (BAX) and BCL2 homologous antagonist/killer (BAK), causing mitochondrial outer membrane permeabilization (MOMP). MOMP results in the release of a range of apoptogenic factors, including cytochrome c, from the intermembrane space of the mitochondria into the cytoplasm. In the cytoplasm, cytochrome c binds to apoptotic protease activating factor 1 (APAF1) to form the apoptosome, which mediates the activation of caspase 9. The extrinsic and intrinsic pathways converge with initiator caspases (e.g., caspase 8, caspase 9) activating executioner caspases (e.g., caspase 3, caspase 7, caspase 6), which mediate cellular destruction.

To date, two distinct but convergent pathways to apoptosis have been identified in vertebrates: the extrinsic pathway and the intrinsic pathway (Figure 1). In contrast to the extrinsic pathway, which is initiated when certain death receptor ligands of the tumor necrosis factor (TNF) family (e.g., first apoptosis signal ligand [FAS-L], TNF) bind to their cognate death receptors (e.g., first apoptosis signal [FAS], TNF receptor [TNFR]) on the plasma membrane, resulting in the activation of caspase 8, the intrinsic pathway is initiated by a diverse range of stress signals (e.g., DNA damage, growth factor deprivation). These stress signals disrupt the integrity of the outer mitochondrial membrane, triggering an event called mitochondrial outer membrane permeabilization (MOMP) [8]. Recognized as the “point of no return”, MOMP causes the release of apoptogenic factors, including cytochrome c, second mitochondria-derived activator of caspase (SMAC) and Omi, from the intermembrane space of the mitochondria into the cytosol [9], where they facilitate the activation of caspase 9 in the apoptosome. The extrinsic and intrinsic pathways converge with initiator caspases (e.g., caspase 8, caspase 9) activating executioner caspases (e.g., caspase 3, caspase 7, caspase 6), which degrade cellular components and prepare dying cells for phagocytosis with minimal disruption to surrounding tissues [10].

### 1.1. BCL2 Family Proteins: The Major Regulators of Apoptosis

Commitment to the intrinsic pathway of apoptosis is governed by the B-cell lymphoma 2 (BCL2) family of proteins, which participate in a complex network of protein–protein interactions to regulate the integrity of the outer mitochondrial membrane. The *BCL2* gene, encoding the founding member of this protein family, was initially identified from the breakpoint region of t(14;18), which is a recurrent chromosomal translocation in follicular lymphoma (FL) [11,12]. Driven by the immunoglobulin heavy chain gene promoter on chromosome 14, the translocated *BCL2* gene becomes overexpressed in FL [13]. Subsequent studies discovered that the enforced expression of BCL2 in hematopoietic cells rendered them resistant to death induced by cytokine deprivation. Intriguingly, the overexpression of BCL2 promoted cell survival by maintaining cells in G0 phase, rather than causing them to proliferate as do other oncogenes [14], and mice harboring the *BCL2* transgene showed lymphocyte accumulation without any propensity for tumorigenesis. However, when BCL2 was expressed with another oncogene *MYC*, marked synergistic effects were observed, resulting in rapid lymphoma development from pre-B and B cells [15]. Together, these findings uncovered the unique role of BCL2 as a promoter of cell survival that enabled nascent neoplastic cells to survive for long enough to gain other oncogenic mutations, facilitating their eventual full malignant transformation.

Since the identification of BCL2, 16 additional BCL2 family proteins have been discovered, which share one or more of the BCL2 homology (BH) domains 1–4. Based on structure and function, members of the BCL2 family are divided into three distinct subgroups: pro-apoptotic effectors containing BH domains 1–3 (e.g., BCL2-associated X protein [BAX], BCL2 homologous antagonist/killer [BAK]), pro-survival proteins containing all BH domains (e.g., BCL2, B-cell lymphoma extra large [BCLxL], BCLw, myeloid cell leukemia 1 [MCL1], BCL2-related protein A1), and pro-apoptotic BH3-only proteins containing only a single BH3 domain (e.g., BCL2-associated death promoter [BAD], BIM, BCL2 interacting killer [BIK], truncated BH3 interacting domain death agonist [t-BID], harakiri [HRK], BCL2 modifying factor [BMF], NOXA, p53 upregulated modulator of apoptosis [PUMA]). In the basal state, BAX mainly exists in the cytosol as an inactive monomer with its transmembrane domain folded into its hydrophobic groove [16], and BAK, while constitutively inserted into the outer mitochondrial membrane [17,18], is likewise kept in an inactive monomeric state through its interaction with another outer mitochondrial protein called voltage-dependent anion-selective channel protein 2 (VDAC2) [19]. The activation of BAX induces the release of its transmembrane domain from its hydrophobic groove, facilitating its translocation to the outer mitochondrial membrane [16]. Similarly, the activation of BAK results in its liberation from VDAC2 [19]. Released BAX and BAK undergo further conformational changes to expose their hydrophobic groove and BH3 domain, which bind to each other across different molecules to facilitate the formation of BAX and BAK oligomers [8]. These oligomers are predicted to form macropores in the outer mitochondrial membrane, mediating MOMP and consigning the cell to apoptotic cell death. However, in the absence of apoptotic signals, BAX and BAK are sequestered by pro-survival proteins, which bind through their hydrophobic groove to the BH3 domains of their pro-apoptotic counterparts, inhibiting their activity [20]. Apoptotic stimuli result in the transcriptional and post-translational induction of pro-apoptotic BH3-only proteins, which carry out their pro-apoptotic function by either neutralizing the pro-survival proteins [21] or directly activating BAX and BAK [22,23,24]. BH3-only proteins bind to pro-survival proteins primarily through the insertion of four hydrophobic residues in its BH3 domain into the P1 to P4 pockets in the hydrophobic grooves of the pro-survival proteins. However, due to subtle differences in their BH3 domains and in the hydrophobic grooves of pro-survival proteins, some BH3-only proteins, such as BIM, PUMA, and t-BID, are able to bind with high affinity to all pro-survival proteins, whereas other BH3-only proteins, such as BAD and NOXA, only bind selectively to certain pro-survival proteins [21]. BAD predominantly binds to BCL2, BCLxL, and BCLw, and NOXA shows strong specificity toward MCL1 and A1, but not the other pro-survival proteins, making them less potent in inducing apoptosis.

### 1.2. Dysregulation of Apoptosis in B-Cell Malignancies

The intrinsic pathway of apoptosis is commonly perturbed in B-cell malignancies, including chronic lymphocytic leukemia (CLL), non-Hodgkin lymphomas (NHL) such as mantle cell lymphoma (MCL), FL and diffuse large B-cell lymphoma (DLBCL), and plasma cell dyscrasias such as multiple myeloma (MM) and Waldenström macroglobulinemia (WM). Several mechanisms have been described, including the overexpression of pro-survival proteins and loss of BH3-only protein function.

#### 1.2.1. Overexpression of Pro-Survival Proteins

High BCL2 expression is almost universal in FL, CLL, MCL, and WM, but it occurs through diverse mechanisms. In FL, BCL2 overexpression is driven by the t(14;18)(q32;q21) translocation, which places the *BCL2* gene on chromosome 18 under the control of the immunoglobulin heavy chain promoter on chromosome 14 [13]. In CLL, deletion of or mutations in the genetic loci for miR-15a and miR-16-1, which negatively regulate BCL2 expression, results in upregulation of BCL2 [25].

In addition to BCL2, MCL1 has been shown to be essential for the sustained expansion of MM [26] and MYC- or BCR-ABL1-driven pre-B or B-cell lymphomas [27,28]. Approximately 40% of patients with MM carry a gain or amplification of 1q21, which is a chromosomal region that contains both the *MCL1* and *IL-6R* genes [29], which is associated with inferior progression-free and overall survival [30]. In DLBCL, high MCL1 expression is seen predominantly in the activated B-cell (ABC) subtype and is driven by recurrent chromosomal gains or amplifications of the MCL1 locus in up to 26% of cases [31].

Finally, elevated BCLxL expression is frequently seen in Burkitt lymphoma, DLBCL, and MM, due to either gene amplification or transcriptional and post-transcriptional regulation [32].

#### 1.2.2. Loss of BH3-Only Protein Function

Homozygous *BIM* deletions have been described in some B-cell lymphomas, including MCL [33,34], and the epigenetic silencing of BIM has been implicated in glucocorticoid resistance in pediatric acute lymphoblastic leukemia (ALL) [35]. However, the dominant mechanism by which the activity of BH3-only proteins is dampened appears to be a loss of TP53 function through the deletion of chromosome 17p (del(17p)) and/or *TP53* mutation, resulting in impairment of TP53-mediated induction of NOXA and PUMA in response to cellular stress, particularly DNA damage [36,37]. Accordingly, in CLL, MM, aggressive lymphomas, and B-lineage ALL, TP53 abnormalities have been associated with poor prognosis and resistance to DNA-damaging chemotherapy.

### 1.3. BH3 Mimetics

**Figure 2:**
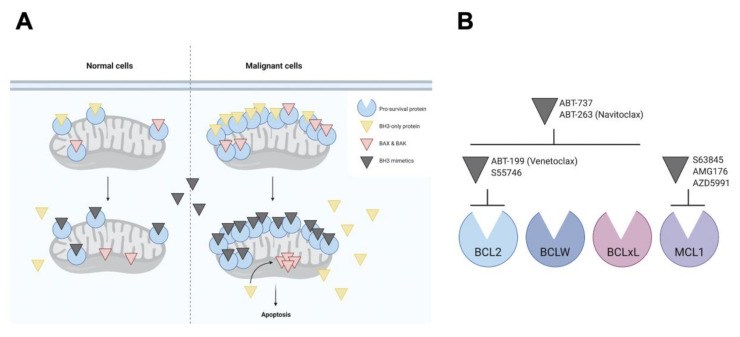
Mechanism of action of BH3 mimetics. (**A**) Pro-survival proteins (e.g., BCL2, B-cell lymphoma extra large [BCLxL], myeloid cell leukemia 1 [MCL1]), shown in blue, are commonly overexpressed in cancer cells, where they sequester high levels of pro-apoptotic proteins, including BH3-only proteins (e.g., BIM), shown in yellow, and pore-forming effector proteins (e.g., BAX, BAK), shown in red, through their BH3 motif to maintain cell survival. These cells are paradoxically “primed” for death, as inhibition of upregulated pro-survival proteins by BH3 mimetics would liberate large quantities of originally sequestered pro-apoptotic proteins, driving the cell toward apoptosis. (**B**) Different BH3 mimetics target different pro-survival proteins.

While cells overexpressing one or more pro-survival members of the BCL2 family have a clear survival advantage, paradoxically, they are also “primed for death”, as inhibition of the upregulated pro-survival proteins would result in the release of large amounts of sequestered pro-apoptotic proteins, driving the cell toward apoptosis (Figure 2A). In other words, the cells become dependent on a specific pro-survival protein for their survival. This concept of “pro-survival addiction” sparked a search for small molecules that could mimic the function of BH3-only proteins in binding with high affinity to the hydrophobic groove of specific pro-survival proteins commonly overexpressed in B-cell malignancies, thereby restoring the capacity of cancer cells to undergo apoptosis. Despite initial doubts as to whether such a broad, hydrophobic protein–protein interface could be “drugged” at all, technological innovations, such as structure–activity relationship (SAR) by nuclear magnetic resonance (NMR) and state-of-the-art combinatorial chemistry, ultimately enabled the design of several potent and specific BH3 mimetics (Figure 2B).

## 2. BCL2 Inhibitors

### 2.1. ABT-737 and ABT-263 (Navitoclax)

The first validated BH3 mimetic was reported in 2005 [38]. ABT-737 bound BCL2, BCLxL, and BCLw with high affinity, thereby mimicking the action of BH3-only protein BAD, demonstrating the sub-micromolar killing of primary cancer cells and cancer cell lines as well as anti-tumor activity in mouse models [38,39]. However, ABT-737 was not sufficiently orally bioavailable to take into clinical trials.

Navitoclax (ABT-263), an orally bioavailable ABT-737 analogue with the same target specificity and activity, was later described in 2008 and was successful in reaching clinical trials [40]. Among several tested B-cell malignancies, navitoclax was most potent against CLL, expressing high levels of BCL2 [40,41]. However, the development of navitoclax was ultimately curtailed by the dose-limiting thrombocytopenia induced by its on-target inhibition of BCLxL, which platelets rely on for their survival [42,43].

### 2.2. Venetoclax (ABT-199)

In an effort to overcome this complication, drug-discovery scientists set out to create a BH3 mimetic that selectively targeted BCL2. The design of a BCL2-selective BH3 mimetic proved challenging due to the high degree of similarity in the BH3 domain of BCL2 and BCLxL. However, using reverse engineering, exploiting subtle differences between the binding interfaces of BCL2 and BCLxL, venetoclax (ABT-199/GDC-0199) was developed in 2013, which bound BCL2 with sub-nanomolar affinity, but interacted weakly with BCLxL and BCLw [44]. In sparing BCLxL, venetoclax had a limited effect on platelets but retained potent single-agent cytotoxic activity in a range of BCL2-dependent tumors, including CLL [44,45].

#### 2.2.1. Venetoclax in CLL

Based on promising preclinical data, a phase 1 first-in-human trial of venetoclax (M12-175; NCT01328626) was conducted in 116 patients with relapsed and/or refractory (R/R) CLL (Table 1), including those harboring disease features associated with poor response to chemoimmunotherapy, such as fludarabine refractoriness, TP53 aberrations (e.g., del(17p), TP53 mutations) and unmutated immunoglobulin heavy chain variable region (IGHV) genes [46]. In two of the first three patients to be dosed with venetoclax, significant reductions in circulating tumor burden and palpable lymphadenopathy were seen within 8 hours and 24 hours, respectively [44]. Despite the majority of patients having received multiple lines of prior therapy, venetoclax proved to be active at all dose levels tested (150–1200 mg/day). The overall response rate (ORR) was 79%, with 20% of patients achieving a complete response (CR) by International Workshop on CLL (iwCLL) criteria. Minimal residual disease (MRD) was evaluated in 17 of the 23 patients who had achieved a CR, and six (35%) were found to be MRD negative, which is defined as less than one CLL cell detected per 10,000 leukocytes analyzed by multiparameter flow cytometry [47].

Remarkably, objective responses were seen in 79% of patients with fludarabine-resistant disease and 71% of patients with del(17p) CLL. These impressive results were consistent with the expected mechanism of action of venetoclax (Figure 2), which predicts that the activity of venetoclax should be independent of the presence of an intact TP53 pathway. Indeed, in vitro and in vivo sensitivity assays confirmed that response to venetoclax was not affected by del(17p), *TP53* mutation, or loss of TP53 function [48]. In the clinical setting, the efficacy of venetoclax monotherapy in TP53-aberrant CLL was confirmed in a subsequent phase 2 trial (M13-982; NCT01889186) (Table 1), in which an ORR of 79.4% was documented amongst 107 patients with R/R CLL who carried del(17p) and had received ≥ 1 prior therapy [49]. Based on these results, venetoclax was granted breakthrough therapy designation by the United States Food and Drug Administration (FDA) in May 2015 and in April 2016 received approval for use in patients with CLL who have del(17p) and have received ≥ 1 prior therapy. A separate phase 2 study evaluated venetoclax monotherapy in 127 patients with R/R CLL after B-cell receptor (BCR) inhibitor ibrutinib or idelalisib treatment (M14-032; NCT02141282) (Table 1) and observed ORRs of 65% and 67% in the ibrutinib- and idelalisib-relapsed patients [50,51], delineating venetoclax as a viable therapeutic option after disease progression on BCR inhibitor therapy.

Although generally well-tolerated, venetoclax is not without toxicities. In a comprehensive safety analysis of venetoclax monotherapy using data from M12-175, M13-982, and M14-032, the most common adverse events of any grade were mild gastrointestinal symptoms and cytopenias [52]. In addition, tumor lysis syndrome (TLS) was identified early in the development of venetoclax as an important risk at drug initiation. Largely a consequence of the exquisite sensitivity of CLL cells to venetoclax, TLS was responsible for the death of two patients in early clinical trials [53]. Subsequent modifications to protocols, including dose escalation, TLS risk stratification, TLS prophylaxis, and close laboratory monitoring, have enabled TLS risk to be effectively mitigated [52].

Notwithstanding the impressive response rates obtained with single-agent venetoclax, preclinical data have strongly suggested that combining venetoclax with other agents may result in synergistic effects [44]. In a phase 1b trial of venetoclax plus the anti-CD20 monoclonal antibody rituximab (VEN+R) in 49 patients with R/R CLL (M13-365; NCT01682616) (Table 1), an ORR of 86% was achieved, including a CR in 51% of patients [53]. Systematic evaluations of serial bone marrow (BM) samples demonstrated achievement of MRD negativity in 57% of all patients. Patients who achieved deep responses (CR or CR with incomplete marrow recovery [CRi] or MRD-negative PR) remained in ongoing remission for periods of up to 2 years after discontinuation of venetoclax therapy, demonstrating the feasibility of time-limited therapy for a subset of patients treated with VEN+R. Based on these results, VEN+R was granted breakthrough therapy designation by the FDA for use in R/R CLL in January 2016. The phase 3 MURANO trial (NCT02005471) compared the efficacy of VEN+R with standard chemoimmunotherapy, bendamustine plus rituximab (BR), in patients with R/R CLL (Table 1) [54]. After a median follow-up of 23.8 months, the rate of progression-free survival (PFS) was significantly higher in the VEN+R group than in the BR group (84.9% vs. 36.3%). Moreover, combination therapy with VEN+R achieved a higher MRD negativity rate than the standard BR regimen (83.5% vs. 23.1%) or any agent or combinations of agents previously evaluated in R/R CLL trials, suggesting that superior efficacy results can be attained by replacing chemotherapy with venetoclax than by adding other targeted agents to chemoimmunotherapy [55]. Not unexpectedly, VEN+R was associated with a higher incidence of grade 3 or 4 neutropenia than BR (57.7% vs. 38.8%), but TLS risk remained low at a rate of 3.1%. The results of the MURANO trial resulted in the approval of venetoclax by the FDA for the treatment of patients with CLL or SLL irrespective of del(17p) status.

Combining venetoclax with the second-generation anti-CD20 antibody obinutuzumab (VEN+G) was shown to enhance cell death in CLL patient samples treated ex vivo [56], which is an observation that has since been recapitulated in the clinic. In the phase 3 CLL14 trial (NCT02242942) comparing the efficacy of VEN+G to chlorambucil–obinutuzumab in the front-line setting (Table 1), the proportion of patients with PFS at 24 months was significantly higher in the VEN+G group than in the chlorambucil–obinutuzumab group (88.2% vs. 64.1%) [57]. Moreover, 49.5% of patients in the VEN+G group achieved a CR, which compares favorably to other therapies frequently used in this setting. The rate of MRD negativity was also higher in the VEN+G arm (75.5% in PB, 56.9% in BM) than in the chlorambucil–obinutuzumab arm (35.2% in PB, 17.1% in BM). Results from the CLL14 trial precipitated at last the approval of venetoclax as a front-line therapy in CLL or SLL in May 2019. The ongoing phase 2 HOVON 139/GIVE trial is currently investigating whether venetoclax-mediated TLS risk can be mitigated in the population unfit for fludarabine-containing chemoimmunotherapy by pre-induction with obinutuzumab and whether MRD-guided duration of venetoclax treatment is a feasible and effective approach [58]. In the reported interim safety analysis and preliminary data of the first 30 patients, pre-induction with obinutuzumab is well-tolerated and resulted in a downgrading of high TLS risk. A high proportion of patients achieved MRD negativity after combination therapy, providing preliminary evidence that this novel regimen utilizing venetoclax and obinutuzumab could result in a very high percentage of MRD negativity with an abrogation of TLS risk.

Aberrant BCR signaling plays an important role in the pathogenesis of CLL. Evidence suggests that the BCR of CLL cells is engaged by autoantigen, resulting in constitutive BCR activation in vivo [59,60,61]. Constitutive BCR signaling results in enhanced activity of kinases downstream of BCR, including phosphatidylinositol 3-kinase (PI3K) and Bruton tyrosine kinase (BTK), and drives not only the autonomous proliferation and survival of tumor cells, but also homing of tumor cells to protective niches in the BM and lymph nodes, providing the preclinical rationale for testing venetoclax in combination with PI3K and BTK inhibitors, which are known to mobilize CLL cells into the peripheral blood (PB) compartment. In BH3 profiling studies, treatment of CLL cells co-cultured with stroma with the PI3K-δ inhibitor idelalisib was shown to cause CLL cell de-adhesion, leading to increased apoptotic priming [62]. Similarly, the dual PI3K-δ and PI3K-γ inhibitor duvelisib was shown in reverse phase protein arrays and immunoblots to increase BCL2 protein expression, increasing the sensitivity of ex vivo-cultured CLL cells obtained from duvelisib-treated patients to venetoclax [63]. Finally, the pharmacological profiling of residual circulating CLL cells from patients being treated with the BTK inhibitor ibrutinib revealed an exquisite dependence on BCL2 and sensitivity to venetoclax, which is likely related to increased mobilization of lymphocytes from lymph nodes and decreased levels of MCL1 and BCLxL following ibrutinib therapy [64]. Recently, the combination of venetoclax and ibrutinib (VEN+IBR) has been evaluated in patients with CLL in both the front-line and R/R settings. In an investigator-initiated phase 2 study of VEN+IBR in 80 older patients with previously untreated, high-risk CLL (Table 1), 88% of patients achieved CR or CRi after 12 cycles of the combination targeted therapy, and 61% attained MRD negativity [65]. The phase 2 CLARITY study combined venetoclax and ibrutinib in the treatment of 53 patients with R/R CLL [66]. After 12 months of VEN+IBR, an ORR of 89% was achieved, with 51% of patients attaining CR. MRD negativity was achieved in the PB of 53% and in the BM of 36%. These results show the potent synergy between venetoclax and ibrutinib for the clearance of MRD, opening up the possibility of limited-duration VEN+IBR therapy for patients with CLL with a durable maintenance of remission.

The capacity of venetoclax-based combination therapy to enhance clinical efficacy has led to the emergence of multiple novel regimens attempting to combine venetoclax with up to two active agents while avoiding a concomitant increase in safety concerns. A phase 1b study (NCT02427451) evaluated a fixed-duration regimen using sequentially administered obinutuzumab followed by VEN+IBR in 12 patients with R/R CLL (Table 1) [67]. The regimen was found to be safe and tolerable. An ORR of 92% was achieved, with 42% attaining a CR or CRi. In a subsequent phase 2 study of the triplet regimen in patients with either treatment-naïve or R/R CLL, a safety profile similar to the phase 1b study was seen [68,69]. Objective responses were reported in all treatment-naïve patients to date, with 50% achieving CR or CRi and 58% achieving MRD negativity in both PB and BM [68]. The ORR of R/R patients at mid-therapy was 92%, with 70% achieving MRD negativity in both PB and BM [69]. Furthermore, sequential treatment with two cycles of bendamustine, followed by induction and maintenance with VEN+G, has been investigated in the phase 2 CLL2-BAG trial (NCT02401503) (Table 1) [70]. The consecutive application of bendamustine and obinutuzumab combined with venetoclax caused no unexpected or cumulative toxicities. Objective responses were seen in 95% of patients at the end of induction, including all treatment-naïve patients and 90% of R/R patients. Longer-term follow-up will likely clarify the durability of responses with these triplet regimens.

#### 2.2.2. Venetoclax in NHL

Given that BCL2 was first discovered as a partner in the t(14;18) translocation that defines FL, venetoclax has also been extensively studied in the context of a range of NHLs. Early preclinical studies on venetoclax had shown potent cytotoxic activity in a subset of NHL cell lines, including not only FL, but also DLBCL and MCL, and these findings were replicated in xenograft models of DLBCL [44] and aggressive progenitor-cell lymphomas derived from bitransgenic MYC/BCL2 mice [73]. In the clinical setting, the initial phase 1 M12-175 trial of venetoclax monotherapy included a cohort of 106 patients with R/R NHL (Table 2) [74]. The trial found venetoclax to be well-tolerated in patients with R/R NHL and to have significant but varied single-agent activity amongst the different NHL subtypes [74]. The highest response rate was seen in MCL (ORR 75%, CR 21%, median PFS 14 months). Significant anti-tumor activity was also observed in FL (ORR 38%, CR 14%, median PFS 11 months) and DLBCL (ORR 18%, CR 12%, median PFS 1 month) [74].

The ideal combination partners for venetoclax in NHL remain unclear, although trials are underway to evaluate the safety and efficacy of combining venetoclax with chemotherapy, monoclonal antibodies, and BCR inhibitors. In the recent phase 2 AIM study, 24 patients with R/R or treatment-naïve MCL were treated with VEN+IBR combination therapy (Table 2) [75]. Dual targeting of BCL2 and BTK resulted in a CR rate of 71%. MRD negativity was documented in 67% of patients according to flow cytometry [75]. The phase 3 SYMPATICO study (NCT03112174) is currently comparing VEN+IBR combination therapy to that of ibrutinib and placebo (Table 2).

The phase 1b CAVALLI trial (NCT02055820) investigated the use of venetoclax in combination with R-CHOP or G-CHOP chemotherapy in 56 patients with NHL, the majority of whom had FL or DLBCL (Table 2) [76]. The ORR was 87.5% for the entire study population, and CR rates of 79.2% and 78.1% were achieved in the venetoclax plus R-CHOP and venetoclax plus G-CHOP arms, respectively [76]. These response rates compared favorably with historical rates from studies such as GOYA, GAUDI, and GALLIUM. The high CR rate of 87.5% in patients with double-expressor (BCL2+ MYC+) DLBCL was a particularly promising finding, and venetoclax plus R-CHOP is currently being evaluated in patients with newly diagnosed DLBCL in the phase 2 portion of the study [76].

#### 2.2.3. Venetoclax in Plasma Cell Dyscrasias

Multiple molecular subtypes of MM have been identified that demonstrate differential expression of pro-survival BCL2 family members [77], with survival dependencies contingent primarily on the distribution of BIM between BCL2/BCLxL and MCL1 [78]. Preclinical studies showed that, similar to ABT-737, venetoclax is particularly active against MM cell lines and patient samples with a t(11;14) translocation and high BCL2/MCL1 mRNA ratios [79]. The clinical efficacy of venetoclax in MM was first demonstrated in a phase 1 study of venetoclax monotherapy in 66 R/R MM patients who had received a median of five prior therapies (NCT01794520) (Table 3) [80]. The trial reported an ORR of 21%, with 15% of patients achieving at least a very good partial response (≥VGPR). Consistent with in vitro studies, the majority of responses (86%) in this phase 1 trial occurred in MM patients with t(11;14). In this group, the ORR was 40%, including 14% CR and 13% VPGR, and the median PFS was 6.6 months.

The activity of single-agent venetoclax in MM is often compromised due to high MCL1 expression. The addition of the corticosteroid dexamethasone to venetoclax was shown to increase venetoclax efficiency in myeloma cell lines by upregulating the expression of BIM and shifting its binding toward BCL2 [81]. Furthermore, the proteasome inhibitor bortezomib was shown to induce apoptosis by stabilizing NOXA, which binds and neutralizes MCL1 [82,83,84]. Following studies in MM xenograft models where bortezomib was shown to enhance venetoclax efficiency, a phase 1b trial (NCT01794507) established the clinical efficacy of venetoclax in combination with bortezomib in the treatment of R/R MM (Table 3) [85]. Amongst 66 patients, an ORR of 67% was achieved, with 42% attaining ≥ VGPR. Based on these impressive results, the efficacy of venetoclax–bortezomib–dexamethasone was compared to that of placebo–bortezomib–dexamethasone in the phase 3 BELLINI trial in patients with R/R MM (NCT02755597) (Table 3) [86]. The addition of venetoclax to bortezomib and dexamethasone conferred a significant PFS benefit (22.9 months vs. 11.4 months). ORR and VPGR rates were also higher in the venetoclax arm compared to the placebo arm (ORR 84% vs. 70%, VPGR 61% vs. 40%). Despite promising PFS and response data, the overall survival findings were marred by an increased number of deaths in the venetoclax arm mainly due to treatment-emergent infections, prompting the FDA to place a partial clinical hold on all trials examining venetoclax in MM in March 2019.

Carfilzomib is a selective, second-generation proteasome inhibitor that demonstrated superior outcome compared to bortezomib for R/R MM patients in the phase 3 ENDEAVOR trial [87,88]. The safety and efficacy of venetoclax in combination with carfilzomib and dexamethasone (VenKd) are currently being assessed in the phase 1/2 M15-538 study (NCT02899052) (Table 3). Preliminary data suggest that VenKd is well-tolerated with promising clinical efficacy [89]. Ixazomib is the first oral proteasome inhibitor to be approved for R/R MM in combination with lenalidomide and dexamethasone [90]. The triple oral combination of venetoclax, ixazomib, and dexamethasone is currently being evaluated in a phase 1/2 study (NCT03399539) (Table 3).

In addition to proteasome inhibitors, venetoclax is also being combined with several monoclonal antibodies in the treatment of patients with MM. Daratumumab is a fully humanized monoclonal antibody targeting CD38, which has been approved for use in R/R MM. The phase 2 M15-654 trial (NCT03314181) is currently evaluating whether daratumumab could increase the anti-tumor activity of venetoclax and venetoclax–bortezomib combination therapy in R/R MM patients (Table 3). Early data demonstrates a tolerable safety profile with encouraging clinical efficacy, with objective responses seen in 92% of patients receiving venetoclax–daratumumab–dexamethasone combination therapy without bortezomib (VenDd) and 88% of patients receiving venetoclax–daratumumab–dexamethasone combination therapy with bortezomib (VenDVd) [91]. Interactions of MM cells with the BM microenvironment result in the dysregulation of several signaling pathways, including the Ras–Raf–MEK–ERK pathway [92]. Cobimetinib is a third-generation MEK inhibitor that has been evaluated in combination with the BRAF inhibitor vemurafenib in R/R extramedullary MM harboring the BRAF V600E mutation [93]. An ongoing phase 1b/2 study (NCT03312530) is assessing the safety and efficacy of cobimetinib monotherapy, cobimetinib plus venetoclax, and cobimetinib plus venetoclax plus an anti-PD-L1 monoclonal antibody atezolizumab in patients with R/R MM (Table 3).

In addition to MM, venetoclax is also being evaluated for use in patients with WM. In a phase 2 trial that included 31 patients with previously treated WM (NCT02677324), treatment with 800 mg of daily venetoclax for a maximum of 2 years resulted in VGPR in six patients (19%), PR in 19 (61%), and a minor response in two (6%) for an ORR of 87% and major response rate of 81% [94]. The 2-year PFS rate was 76%. Grade 4 neutropenia occurred in five patients and grade 3 adverse events included neutropenia in 15 patients, anemia in four patients, and diarrhea in four. One instance of laboratory TLS occurred but no immunoglobulin M (IgM) flare, clinical TLS, or deaths were observed over a median follow-up of 18 months.

### 2.3. Resistance to BCL2 Inhibitors

Despite the impressive clinical efficacy of venetoclax across a range of B-cell malignancies, resistance to venetoclax occurs in a subset of patients and is an emerging therapeutic challenge. Venetoclax resistance can manifest either as the primary failure of certain tumor subtypes to respond to venetoclax or as acquired resistance driving disease progression on venetoclax therapy following an initial response [95].

#### 2.3.1. Primary Resistance to Venetoclax

Early trials of single-agent venetoclax found that sensitivity to venetoclax varies greatly amongst different subtypes of B-cell malignancies. While the ORR of venetoclax monotherapy in CLL and MCL was high at 79% and 75% respectively, the ORR in FL, MM, and DLBCL was much lower at 38%, 21%, and 18%, respectively [46,74]. Preclinical studies have demonstrated a correlation between in vitro sensitivity to venetoclax and the relative expression of BCL2 to MCL1, BCLxL, or BIM in MCL [96], FL [97], and MM [79]. Whole-exome sequencing of tumor samples from the five MCL patients in the phase 2 AIM trial of VEN+IBR who failed to respond to the combination therapy revealed recurrent genomic abnormalities affecting the SWItch/Sucrose Non-Fermentable (SWI–SNF) chromatin-remodeling complex, including loss of chromosome 9p containing *SMARCA2* in four out of the five patients, pathologic mutations or deletions in *ARID2* in three patients, and mutations in the helicase domain of *SMARCA4* in three patients [98]. The functional impairment of SWI–SNF through loss of *SMARCA4* was shown to result in a dramatic reduction in chromatin accessibility at the locus of ATF3, a direct repressor of BCLxL transcription [99], leading to the downregulation of ATF3 and subsequent overexpression of BCLxL [98].

In addition to genomic abnormalities, soluble factors present in the tumor microenvironment, including CD40L and certain cytokines (e.g., IGF-1, BAFF, IL-6, IL-10), appear to be able to reduce the dependence of certain B-cell malignancies on BCL2 by upregulating one or more alternative pro-survival proteins [96,100,101,102]. Combining venetoclax with a BTK inhibitor, an anti-CD20 monoclonal antibody, or a dual SYK/JAK inhibitor has shown promise in being able to overcome microenvironment-mediated resistance in CLL and MCL [103,104].

#### 2.3.2. Acquired Resistance to Venetoclax

Following a deep and durable response to venetoclax, a proportion of patients develop disease progression while on venetoclax therapy. The time to disease progression varies amongst different subtypes of B-cell malignancies. In patients with del(17p) CLL, approximately 50% relapse after 2 years on venetoclax monotherapy, highlighting the significant challenge that secondary resistance poses [71].

The mechanisms of acquired resistance to venetoclax are still the subject of ongoing research but are known to differ depending on tumor histology. In patients with CLL, the most common recurrent mechanism of venetoclax resistance identified to date is the acquisition of a point mutation in the *BCL2* gene, resulting in the substitution of a valine for glycine at position 101 of the BCL2 protein (G101V mutation) [105]. The emergence of the BCL2 G101V mutation during venetoclax treatment was detected initially in seven out of 15 patients enrolled in venetoclax trials with R/R CLL [105]. Interrogation of the structure of the mutant BCL2 protein revealed that the G101V mutation displaces the adjacent E152 residue into the base of the P2 hydrophobic pocket [106], disrupting the anchoring of venetoclax to BCL2 and causing an approximately 180-fold reduction in the binding affinity [105].

A subsequent study by Tausch and colleagues independently reported the emergence of the BCL2 G101V mutation and identified a second BCL2 D103Y mutation in one of the venetoclax-resistant patients [107]. Recently, multiple other novel BCL2 mutations acquired in parallel with BCL2 G101V mutation have been reported in patients with CLL progression, including D103E, D103V, V156D, R107_R110dup, A113G, and R129L [108]. However, the fact that these variants have only been detected mostly at low sub-clonal frequencies and in only a subset of patients suggests the presence of other acquired resistance mechanisms, which is a hypothesis that is further supported by the failure of two other whole-exome sequencing studies to detect any mutations in BCL2 in patients progressing on venetoclax [109,110].

**Figure 3:**
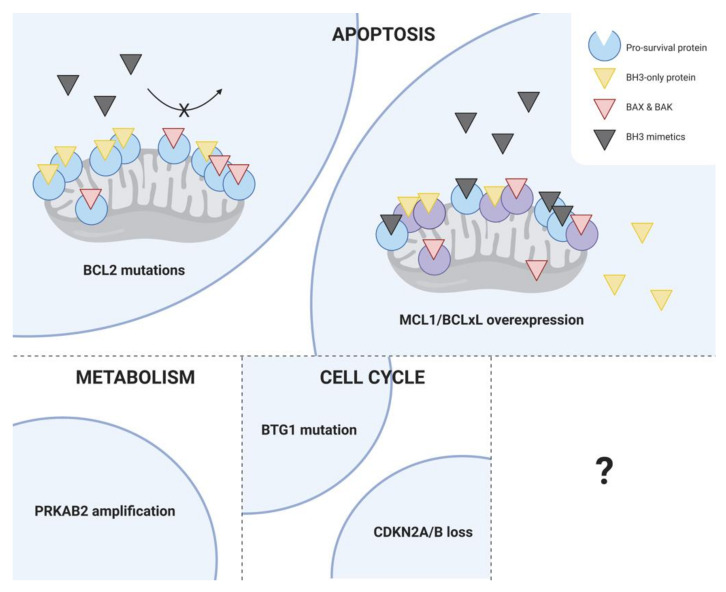
Mechanisms of acquired resistance to venetoclax in CLL. Although the factors contributing to disease progression on venetoclax are still the subject of ongoing research, available studies have identified a diverse range of mechanisms, which broadly target three cellular processes: apoptosis, metabolism, and the cell cycle. The most common recurrent mechanism of venetoclax resistance identified in CLL to date is the acquisition of the BCL2 G101V mutation, although other BCL2 mutations have also been detected (e.g., D103Y, D103E, D103V, V156D, R107_R110dup, A113G, R129L). Other mechanisms include the upregulation of alternative pro-survival proteins, particularly MCL1 and BCLxL, amplification of a positive regulatory subunit of AMPK called PRKAB2, leading to increased oxidative phosphorylation, homozygous deletions of *CDKN2A/B*, and *BTG1* mutations.

Unsurprisingly, the upregulation of alternative pro-survival proteins, in particular MCL1 and BCLxL, has been observed in CLL patients with acquired resistance to venetoclax. In one of the CLL patients with the BCL2 G101V mutation, increased BCLxL protein expression was detected in subclones without the BCL2 G101V mutation [105]. Moreover, amplification of a region on chromosome 1q23 containing MCL1 was recently identified in samples from CLL patients relapsing after venetoclax therapy [109]. Co-located with MCL1 on the amplified region of chromosome 1q23 is a positive regulatory subunit of AMP-activated protein kinase (AMPK) called PRKAB2, and there is evidence to suggest that the amplification of PRKAB2 may lead to enhanced activation of AMPK, resulting in increased oxidative phosphorylation that protects cells against the effect of venetoclax on the electron transport chain [109]. Other contributors of acquired resistance to venetoclax in CLL have been identified using whole-exome sequencing of samples from CLL patients relapsing early during venetoclax therapy, including homozygous deletions of *CDKN2A/B* and *BTG1* mutations [110]. These mechanisms are summarized in Figure 3.

Early in vitro studies using a murine MCL cell line observed the development of two BCL2 mutations, F101L and F101C, which are equivalent to F104L and F104C in humans [111]. In a model of MCL, resistance to venetoclax was observed to evolve from outgrowth of “persister” clones displaying a loss of 18q21 amplicons harboring BCL2 as well as adaptive super enhancer-driven transcriptional reprogramming [112]. However, to date, none of these mechanisms have been seen in patients with MCL treated with venetoclax. Recently, whole-exome sequencing of biopsy samples from seven patients with multiply relapsed MCL who received venetoclax-based therapies has revealed that unlike in CLL, BCL2 mutations are infrequent in venetoclax-resistant MCL, occurring in only one patient at progression; instead, the acquisition of non-BCL2 mutations, including alterations in *TP53*, *SMARC4*, *CELSR3*, *CCND1* and *KMT2D*, may play a role in disease progression [113]. In FL, venetoclax resistance has been studied in t(14;18) positive cell lines and increased phosphorylation of extracellular signal-regulated kinase 1/2 (ERK1/2) and decreased levels of BIM were seen in cell populations that survived venetoclax treatment [97]. However, the only putative mechanism of acquired venetoclax resistance reported in patients with FL to date is the acquisition of the BCL2 F104I mutation, which similar to the BCL2 G101V mutation results in an approximately 300-fold decrease in the affinity of venetoclax for BCL2 [114]. Further studies will likely help clarify the extent to which each mechanism contributes to disease progression on venetoclax and uncover novel determinants that will help to guide therapeutic selection.

## 3. MCL1 Inhibitors

Given the established role of MCL1 as a critical pro-survival factor in a range of hematologic malignancies, including MM, AML, and NHL [26,27,115,116,117], and a resistance factor to BH3 mimetics targeting BCL2 and BCLxL [118], there has been longstanding interest in designing potent and selective MCL1 inhibitors for therapeutic use. However, compared to BCL2 and BCLxL, MCL1 has proven to be more challenging to target, partly because its binding pocket is shallower and more rigid. Moreover, MCL1 has been shown in murine knockout models to play a critical role in the survival of a range of normal tissues, including cardiomyocytes [119,120], hematopoietic stem cells [121], developing and mature lymphocytes [122,123,124,125,126], and oocytes [127], leading to significant concerns around the potential toxicities associated with MCL1 inhibition. Despite this, progress on the development of selective MCL1 inhibitors has accelerated over recent years, and several compounds targeting MCL1 have now entered clinical trials, including AZD5991 [128], S64315/MIK665, AMG-176 [129], and AMG-397 (Table 4).

### 3.1. AZD5991

AZD5991 is a rationally designed macrocyclic molecule with high selectivity and affinity for MCL1 [128]. in vivo, AZD5991 showed potent anti-tumor activity with complete tumor regression in several mouse and rat xenograft models of MM and AML after a single tolerated intravenous injection [128]. The activity of AZD5991 against MM and AML subcutaneous tumors was enhanced when the compound was administered in combination with bortezomib and venetoclax, respectively [128]. Based on these promising data, the clinical efficacy of AZD5991, administered intravenously every 21 days for nine cycles, is currently being evaluated in a phase 1 trial involving patients with R/R hematologic malignancies (NCT03218683) (Table 4).

### 3.2. S63845 and S64315/MIK665

S63845 is a highly potent MCL1 inhibitor that has exhibited impressive anti-tumor activity across a range of MCL1-dependent cancers, including MM and NHL [130]. Notably, in the context of MM, in contrast to the BCL2 inhibitor venetoclax [79], S63845 showed significant activity not only in MM cell lines carrying the t(11;14) translocation but also in those carrying genetic lesions associated with poor prognosis, including t(4;14) and *TP53* mutations, suggesting that MCL1 inhibitors may be effective in cases of MM refractory to standard chemotherapy [130]. Despite concerns regarding the important pro-survival role of MCL-1 in normal tissues, little toxicity was observed in mice treated with S63845 at doses capable of ablating mouse lymphoma cells [130]. Even though S63845 binds to human MCL-1 protein with six-fold higher affinity than it does to mouse MCL-1 protein, studies using a humanized *Mcl-1* mouse model suggest that a therapeutic window could well be established and that drug-mediated inhibition has important phenotypic differences to genetic deletion of the *Mcl-1* locus [131].

S64315/MIK665 is a compound closely related to S63845, for which minimal data have so far been disclosed. A phase 1 study is currently underway to assess the tolerability and preliminary efficacy of the related compound S63415 administered intravenously as a single agent in patients with MM (NCT02992483) (Table 4).

### 3.3. AMG-176 and AMG-397

Optimization of a series of spiromacrocyclic molecules using structure-based design and conformation restriction as guiding principles led to the discovery of a highly selective, orally bioavailable MCL1 inhibitor called AMG-176 [129]. The tool compound AM-8621, which was used to characterize the mechanism of action of AMG-176, has shown a promising effect in vitro. Among hematologic malignancies, MM, AML, and B-cell lymphoma demonstrated the greatest sensitivity to AM-8621 [129]. Given its superior pharmacokinetic properties over AM-8621, the efficacy of AMG-176 was further tested in murine models of MM and AML, and it was found to result in a significant reduction of tumor burden in a dose-dependent manner [129]. Synergistic effects could be achieved through the combination of AMG-176 with venetoclax in AML tumor models and in primary patient samples at a tolerated dose, highlighting the potential for dual MCL1 and BCL2 inhibition [129].

The safety and efficacy of AMG-176 are currently being evaluated both as monotherapy in patients with R/R MM and AML (NCT02675452) and in combination with venetoclax in patients with AML and NHL (NCT03797261) (Table 4). Although administered orally during in vivo studies, AMG-176 is being administered as an intravenous infusion in these clinical trials. The first oral MCL1 inhibitor to reach the clinic is a compound related to AMG-176 called AMG-397 [132]. The phase 1 trial assessing the safety, tolerability, pharmacokinetics, and efficacy of AMG-397 in patients with MM, NHL, and AML (NCT03465540) (Table 4) was placed on hold by the FDA in September 2019 after the discovery of a safety signal for cardiac toxicity, prompting Amgen, the drug manufacturer, to voluntarily halt the phase 1 studies of AMG-176 as a precaution, emphasizing the importance of clinical trials in establishing whether a wide enough therapeutic window can be found for MCL1 inhibitors to allow the use of this novel subclass of BH3 mimetics in patients.

## 4. BCLxL Inhibitors

Despite early studies establishing the indispensable role of BCLxL in maintaining platelet viability [42], interest has remained in developing BH3 mimetics that selectively target BCLxL. Somatic amplification of BCLxL has previously been identified as a mechanism used by certain cancers to promote cell survival [133]. In addition, BCLxL has been strongly implicated as a chemoresistance factor [134], including to venetoclax, opening up the prospect of dual BCLxL and BCL2 inhibition to overcome venetoclax resistance. Finally, it is thought that the capacity of BCLxL inhibitors to suppress platelets could potentially be leveraged to treat myeloproliferative disorders characterized by malignant thrombocytosis. In recent years, several highly potent and selective BCLxL inhibitors have been described, including WEHI-539 [135], A-1155463 [136], and A-1331852 [137]. However, clinical progression of these agents has been challenging, largely due to their on-target effects on normal cells.

The first truly specific synthetic BCLxL inhibitor to be reported was WEHI-539 [135]. In contrast to ABT-737, WEHI-539 exhibited > 400-fold higher affinity for BCLxL than other pro-survival BCL2 family members [135]. As anticipated, WEHI-539 induced the rapid killing of isolated platelets in a caspase-dependent manner [135]. However, the utility of WEHI-539 as a tool compound was ultimately limited by unfavorable physicochemical properties that made in vivo dosing untenable [136]. Using NMR fragment screening, two BCLxL inhibitors were identified that were suitable for in vivo use: A-1155463 and its orally bioavailable relative A-1331852 [136,137]. A-1155463 demonstrated enhanced activity against BCLxL-dependent cell lines compared to WEHI-539, without possessing any of its pharmaceutical liabilities [136]. The intraperitoneal administration of A-1155364 produced reversible thrombocytopenia in non-tumor-bearing mice, and a modest reduction in tumor growth in small cell lung cancer xenografts [136]. Similarly, A-1331852, which has cellular activity 10-fold more potent than A-1155463, induced significant tumor regression as a single agent in ALL xenografts, and it potentiated the effects of docetaxel in subcutaneous xenograft models of solid tumors [137]. Despite promising in vivo activity, neither of these compounds have yet made it to the clinic.

## 5. Conclusions and Future Directions

BH3 mimetics are a novel class of therapeutics that is assuming an increasingly prominent role in the armamentarium against B-cell malignancies. So far, only the selective BCL2 inhibitor venetoclax has been approved for use in patients. The clinical success of venetoclax in treating CLL and AML has sparked enthusiasm to explore other indications for this agent and develop additional BH3 mimetics that can specifically target other pro-survival BCL2 family proteins. Several potent MCL1-specific inhibitors are currently being scrutinized in clinical trials for the treatment of MM and NHL, and selective BCLxL inhibitors have been designed showing significant anti-tumor activity both in vitro and in vivo.

Despite the promise that BH3 mimetics have shown, a number of key challenges remain. Firstly, only a relatively small number of B-cell malignancy subtypes have so far demonstrated clinically important sensitivity to BH3 mimetics as single agents. Current efforts to enhance the efficacy of BH3 mimetics in the clinic have predominantly focused on combining BH3 mimetics with other active agents. Clinical trials have so far confirmed the viability of combining venetoclax with conventional chemotherapeutics, monoclonal antibodies, kinase inhibitors, and proteasome inhibitors. Preclinical studies are elucidating the potential of combining histone deacetylase (HDAC) inhibitors and hypomethylating agents with venetoclax in MM and DLBCL [138,139]. Moreover, a high-throughput screen identified several new kinase inhibitors that may improve venetoclax therapy in R/R CLL in a personalized manner [102].

Another challenge for the further application of BH3 mimetics is on-target toxicity, especially for MCL1 and BCLxL inhibitors. Extensive preclinical tests have suggested that a therapeutic window could be established for MCL1 inhibitors, which has led to the entry of three MCL1 inhibitors into clinical trials. Data on the potential of these drugs are eagerly awaited. No BCLxL inhibitor has successfully entered clinical trials so far due to dose-limiting thrombocytopenia. Multiple innovative strategies are being explored to reduce the toxicity of BH3 mimetics, including conjugating them to antibodies directed specifically at cancer cells [140], encapsulating them in tumor-targeting nanoparticles [141], and converting them into proteolysis-targeting chimeras (PROTACs). Recently, a BCLxL-specific PROTAC created from navitoclax that targets BCLxL to the Von Hippel-Lindau (VHL) E3 ligase for degradation was shown to be able to selectively kill various BCLxL-dependent cancer cells with higher potency than venetoclax but significantly lower platelet toxicity due to minimal expression of VHL in platelets [142].

Finally, the mechanisms of drug resistance to BH3 mimetics appear complex and are yet to be fully delineated. A comprehensive understanding of determinants of venetoclax resistance will be necessary to inform optimal therapeutic strategies following venetoclax failure. The incorporation of correlative biological analyses linked to detailed genomic profiling of patient samples are therefore likely to become increasingly valuable for the identification of biomarkers to predict response in individual patients to this highly promising new class of therapeutics.

## Figures and Tables

**Table 1:** Selected clinical trials of venetoclax in chronic lymphocytic leukemia (CLL).

ClinicalTrials.Gov Identifier	Other Study ID Numbers	Intervention	Disease	Study Phase(s)	Publications
**VEN monotherapy**					
NCT01328626	M12-175	VEN	R/R CLL	1	[46]
NCT01889186	M13-982	VEN	R/R CLL with del(17p)	2	[49,71]
NCT02141282	M14-032	VEN	R/R CLL after BCR inhibitor therapy	2	[50,51]
**VEN combination therapy**					
NCT01682616	M13-365	VEN + R	R/R CLL/SLL	1b	[53]
NCT02005471	GO28667, MURANO	VEN + R	R/R CLL	3	[54,72]
NCT02242942	BO25323, CLL14	VEN + G	CLL	3	[57]
NCT02756897	2015-0860	VEN + IBR	R/R CLL	2	[65]
NCT02427451	OSU-14266, NCI-2015-00252	VEN + IBR + G	R/R CLL	1b/2	[67]
NCT02401503	CLL2-BAG, 2014-000580-40	Bendamustine + VEN + G	CLL	2	[70]

**Table 2:** Selected clinical trials of venetoclax in non-Hodgkin lymphomas (NHLs).

Clinicaltrials.Gov Identifier	Other Study ID Numbers	Intervention	Disease	Study Phase(s)	Publications
**VEN monotherapy**					
NCT01328626	M12-175	VEN	R/R NHL (including MCL, FL, DLBCL, RT-DLBCL, WM, and MZL)	1	[74]
**VEN combination therapy**					
NCT02471391	14/148, AIM	VEN + IBR	MCL	2	[75]
NCT03112174	PCYC-1143-CA, SYMPATICO	VEN + IBR	MCL	3	N/A
NCT02055820	GO27878, 2013-003749-40, CAVALLI	VEN + R-/G-CHOP	NHL (including FL and DLBCL)	1b/2	[76]

**Table 3:** Selected clinical trials of venetoclax in multiple myeloma (MM).

Clinicaltrials.Gov Identifier	Other Study ID Numbers	Intervention	Disease	Study Phase(s)	Publications
**VEN monotherapy**					
NCT01794520	M13-367, 2012-000589-38	VEN	R/R MM	1/2	[80]
**VEN combination therapy**					
NCT01794507	M12-901, 2011-004626-10	VEN + bortezomib + dexamethasone	R/R MM	1	[85]
NCT02755597	M14-031, 2015-004411-20	VEN + bortezomib + dexamethasone	R/R MM	3	[86]
NCT02899052	M15-538	VEN + carfilzomib + dexamethasone	R/R MM	2	[89]
NCT03399539	MC168C, NCI-2017-02456	VEN + ixazomib citrate + dexamethasone	R/R MM	1/2	N/A
NCT03314181	M15-654, 2017-002099-26	VEN + daratumumab + dexamethasone +/- bortezomib	R/R MM	2	[91]
NCT03312530	BO39813, 2017-000830-68	Cobimetinib + VEN +/- atezolizumab	R/R MM	1b/2	N/A

**Table 4:** Clinical trials of MCL1 inhibitors.

Clinicaltrials.Gov Identifier	Other Study ID Numbers	Intervention	Route of Administration	Disease	Study Phase(s)
NCT03218683	D6910C00001	AZD-5991	IV	R/R hematologic malignancies	1
NCT02992483	CMIK665 × 2101, 2016-003624-22	MIK665	IV	R/R MM, R/R NHL	1
NCT02675452	20150161, 2015-004777-32	AMG-176	IV	R/R MM	1
NCT03465540	20170173	AMG-397	PO	R/R MM, R/R NHL	1
